# The colors of our brain: an integrated approach for dimensionality reduction and explainability in fMRI through color coding (i-ECO)

**DOI:** 10.1007/s11682-021-00584-8

**Published:** 2021-10-24

**Authors:** Livio Tarchi, Stefano Damiani, Paolo La Torraca Vittori, Simone Marini, Nelson Nazzicari, Giovanni Castellini, Tiziana Pisano, Pierluigi Politi, Valdo Ricca

**Affiliations:** 1 Psychiatry Unit, Department of Health Sciences, University of Florence, viale della Maternità, Padiglione 8b, AOU Careggi, Firenze, Florence, FI 50134 Italy; 2 Department of Brain and Behavioral Sciences, University of Pavia, Pavia, PV Italy; 3 Department of Epidemiology, University of Florida, Gainesville, FL USA; 4 Council for Agricultural Research and Economics (CREA), Research Centre for Fodder Crops and Dairy Productions, Lodi, LO Italy; 5 Pediatric Neurology, Neurogenetics and Neurobiology Unit and Laboratories, Neuroscience Department, Meyer Children’s Hospital, University of Florence, Florence, Italy

**Keywords:** fMRI, ReHo, Eigenvector Centrality, fALFF, Psychiatry

## Abstract

Several systematic reviews have highlighted the role of multiple sources in the investigation of psychiatric illness. For what concerns fMRI, the focus of recent literature preferentially lies on three lines of research, namely: functional connectivity, network analysis and spectral analysis. Data was gathered from the UCLA Consortium for Neuropsychiatric Phenomics. The sample was composed by 130 neurotypicals, 50 participants diagnosed with Schizophrenia, 49 with Bipolar disorder and 43 with ADHD. Single fMRI scans were reduced in their dimensionality by a novel method (i-ECO) averaging results per Region of Interest and through an additive color method (RGB): local connectivity values (Regional Homogeneity), network centrality measures (Eigenvector Centrality), spectral dimensions (fractional Amplitude of Low-Frequency Fluctuations). Average images per diagnostic group were plotted and described. The discriminative power of this novel method for visualizing and analyzing fMRI results in an integrative manner was explored through the usage of convolutional neural networks. The new methodology of i-ECO showed between-groups differences that could be easily appreciated by the human eye. The precision-recall Area Under the Curve (PR-AUC) of our models was > 84.5% for each diagnostic group as evaluated on the test-set – 80/20 split. In conclusion, this study provides evidence for an integrative and easy-to-understand approach in the analysis and visualization of fMRI results. A high discriminative power for psychiatric conditions was reached. This proof-of-work study may serve to investigate further developments over more extensive datasets covering a wider range of psychiatric diagnoses.

**Supplementary Information:**

The online version contains supplementary material available at 10.1007/s11682-021-00584-8.

## Introduction

Several systematic reviews have highlighted the role of multiple sources in the investigation of psychiatric illness (Keshavan et al., 2020). In particular, for what concerns functional Magnetic Resonance Imaging (fMRI), the focus of recent literature lies on three lines of research, namely functional connectivity (Damiani et al., 2021; Du et al., 2018; Giraldo-Chica et al., 2018; Sheffield & Barch, 2016; Sörös et al., 2019; L. Zhang et al., 2020; Y. Zhou et al., 2015), network analysis (Jiang et al., 2019; Scalabrini et al., 2020; M. Zhou et al., 2019a, 2019b; Q. Zhou et al., 2017; Y. Zhou et al., 2007), and spectral analysis (Malhi et al., 2020; Shang et al., 2016; P. Zhang et al., 2018; C. Zhou et al., 2019a, b).

Functional Connectivity in fMRI mainly stresses two different phenomena during image acquisition. The first is the long-distance relationship between brain areas, with one main region serving as a seed or reference. The second phenomenon is local connectivity between a brain region and its neighborhood, which can be measured by Regional Homogeneity – ReHo (Zang et al., 2004).

Measures of centrality in fMRI derive from graph-based analyses and are considered a computationally efficient tool for capturing intrinsic neural networks architecture in the human brain (Achard et al., 2006; He et al., 2009; Sporns et al., 2007). In this study, we investigated Eigenvector Centrality—ECM (Lohmann et al., 2010), as other commonly used centrality measurements (e.g. Degree of Centrality) are more sensitive to higher order cortical regions and less sensitive to subcortical ones (Zuo et al., 2012). As recent research in fMRI has shifted attention from cortical to subcortical areas in the investigation of psychiatric disorders (Damiani et al., 2020; Giraldo-Chica & Woodward, 2017; Lottman et al., 2019; Q. Zhou et al., 2017), ECM was preferred.

Spectral analyses in fMRI are based on the notion that valuable information can be found analyzing results in a time-domain manner, as opposed to the more commonly used space-domain. In our spectral analyses, we used fractional Amplitude of Low-Frequency Fluctuations—fALFF (Zou et al., 2008). Recent research has focused on fALFF as one of the most promising parameters for detecting regional signals change in relation to spontaneous activity (F. Liu et al., 2013; Yu-feng et al., 2007).

The technical barrier between the neuroimaging field and clinical practice may delay the transition of analytic results from the overall scientific debate to professional applications. Therefore, the present study aimed to offer a novel method to aid in analyzing, reporting, and visualizing fMRI results in a structured and integrated manner. A more accessible method to analyze and report fMRI results could support both research and clinical practice, as well as benefitting rapid fruition in a dual manner: through numerical dimensionality reduction for machine-learning potentials, through color-coding for human readability. The authors refer to this new proposed methodology by its acronym i-ECO (integrated-Explainability through Color Coding).

### Aims of the study

The purpose of the present study was to evaluate a novel method of visualizing and interpreting fMRI results, based on the integration between functional connectivity, network analysis and time-domain analyses. The primary endpoint was to report the results of the proposed novel method of visualization. The secondary endpoint was to observe the discriminative power of the proposed novel method in the classification of participants based on their psychiatric clinical status.

## Materials and methods

### Study design

The present study was conducted on a shared neuroimaging dataset from the UCLA Consortium for Neuropsychiatric Phenomics, which included imaging and clinical data for 130 healthy adults, men or women between 21 and 50 years old. The shared dataset also included 50 participants diagnosed with Schizophrenia, 49 participants diagnosed with Bipolar disorder and 43 participants diagnosed with ADHD. Diagnoses were reached following DSM-IV TR criteria (American Psychiatric Association, 2000), through the Structured Clinical Interview for DSM-IV, SCID-I (American Psychiatric Association, 2000), in addition to a structured interview for Adult ADHD derived from the Kiddie Schedule for Affective Disorders and Schizophrenia, Present and Lifetime Version (Ambrosini et al., 1989; Poldrack et al., 2016; Schmidt et al., 2013). Further details about the sample can be found in the original study (Poldrack et al., 2016).

### Sample—procedures

fMRI data preprocessing steps were implemented in AFNI (http://afni.nimh.nih.gov/afni) (Cox, 1996; Cox & Hyde, 1997; Taylor et al., 2018). Firstly, the structural and functional reference images were co-registered (Saad et al., 2009). The first 4 frames of each fMRI run were removed in order to discard the transient effects in amplitude observed until magnetization achieves steady state (Caballero-Gaudes & Reynolds, 2017). Slice timing correction (Konstantareas & Hewitt, 2001) and despike methods (Satterthwaite et al., 2013) were applied. Rigid-body alignment of the structural and functional image was performed. The anatomical image was then warped using the Montreal Neurological Institute standard space (MNI152_T1_2009c) template provided with the AFNI binaries. Volume registration was then used to align the functional data to the base volume, warping it to the stereotactic space of choice. Spatial blurring was performed, with a kernel of full width at half maximum of 6 mm. Bandpass (0.01–0.1 Hz) was performed (William R. Shirer et al., 2015). Each of the voxel time series was then scaled to have a mean of 100. To control for non-neural noise, regression based on the 6 rigid body motion parameters and their derivates was applied, as well as mean time series from cerebro-spinal fluid masks (Fox et al., 2005; Vovk et al., 2011) eroded by one voxel (Chai et al., 2012). Regression of white matter artefacts was performed through the fast ANATICOR technique as included in AFNI (Jo et al., 2010). To further improve motion correction, censoring of voxels with a Framewise Displacement above 0.5 mm was applied (Power et al., 2014) to the timeseries in network and functional connectivity analyses, while the time-domain analyses used non-censored data in order to preserve continuity along the time axis.

Subjects with excessive motion were excluded (> 2 mm of motion and/or more than 20% of timepoints above FD 0.5 mm). Overall, 44 subjects were excluded from the fMRI analysis: 11 Neurotypicals, 18 participants with Schizophrenia, 11 subjects with a diagnosis of Bipolar Disorder, and 4 with a diagnosis of ADHD.

### Primary aim—methods

The ReHo value was calculated to measure the similarity of the time series of a given voxel to its nearest 26 voxels (Taylor & Saad, 2013; Zang et al., 2004). In each participant, the Kendall’s Coefficient of Concordance (KCC) for each voxel was normalized using Fisher z-transformation with the formula:

$$\stackrel{\sim }{\rho }=\frac{1}{2}ln\frac{\left(1+\rho \right)}{\left(1-\rho \right)}$$

where $\stackrel{\sim }{\rho }$ represents the normalized value of ρ, the voxel’s KCC value.

The ECM value was calculated through the Fast Eigenvector Centrality method as described by Wink et al. (Wink et al., 2012). 13 Neurotypicals, 5 participants with Schizophrenia, 7 participants with Bipolar Disorder and 4 participants with ADHD were excluded as at least one region had an ECM value of 0, as it was not possible to calculate their ECM value due to computational or technical impossibility to determine result matrices from the data structure (Wink et al., 2012).

The fALFF value was calculated by FATCAT functionalities (Taylor & Saad, 2013) in order to estimate spectral parameters. Firstly, data was bandpassed and the time series average, as well as the Nyquist frequency, were excluded. After the exclusion of selected frequencies and performing a bandpass, the time series was transformed in a periodogram using a Fast Fourier Transform (FFT). The frequency domain thus was in the range from 1/T to the Nyquist frequency, where T was the total duration of the time series. The step size between frequencies was given by the sampling time (1/TR), where TR was the repetition time or the length in time between two consecutive points on a repeating series of acquisitions.

By calculating the voxel-wise values, individual variations were summarized by averaging results per Region of Interest (ROI). For each participant, average values per functional network were obtained. Reference network masks were retrieved from the Functional Imaging in Neuropsychiatric Disorder Lab website – University of Stanford (Greicius & Eger, 2021; W. R. Shirer et al., 2012) and referred to as Regions of Interests (ROIs). Individual values were then scaled through the following formula:

$$\tilde{x }=255\times \frac{x-min}{max-min}$$

where $\tilde{x }$ represents the scaled value of x, the individual subject’s value, and *max* and *min* represented respectively the overall maximum and minimum value per subject, per variable, per ROI.

The ECM, ReHo and fALFF values, per subject, per ROI, were then condensed through a color mixing technique using an additive color model (RGB). ECM values were interpreted as the red component, fALFF as the green component and ReHo as the blue component.

Images for each subject were then compiled through *Python 3.8.5* (Van Rossum & Drake, 2009) and the following libraries: *numpy* (Harris et al., 2020), *PIL* (Umesh, 2012)*.* Average images per diagnostic group were drawn by averaging values by group and compiling the resulting image. Images obtained by subtracting resulting images per diagnostic group versus neurotypicals were drawn. An heatmap describing the numerical differences in scaled feature values was plotted. A MANOVA test for each feature (ECM, fALFF, ReHo) was carried forward, with the diagnostic labels as fixed factors.

### Secondary aim—methods

**Fig. 1:**
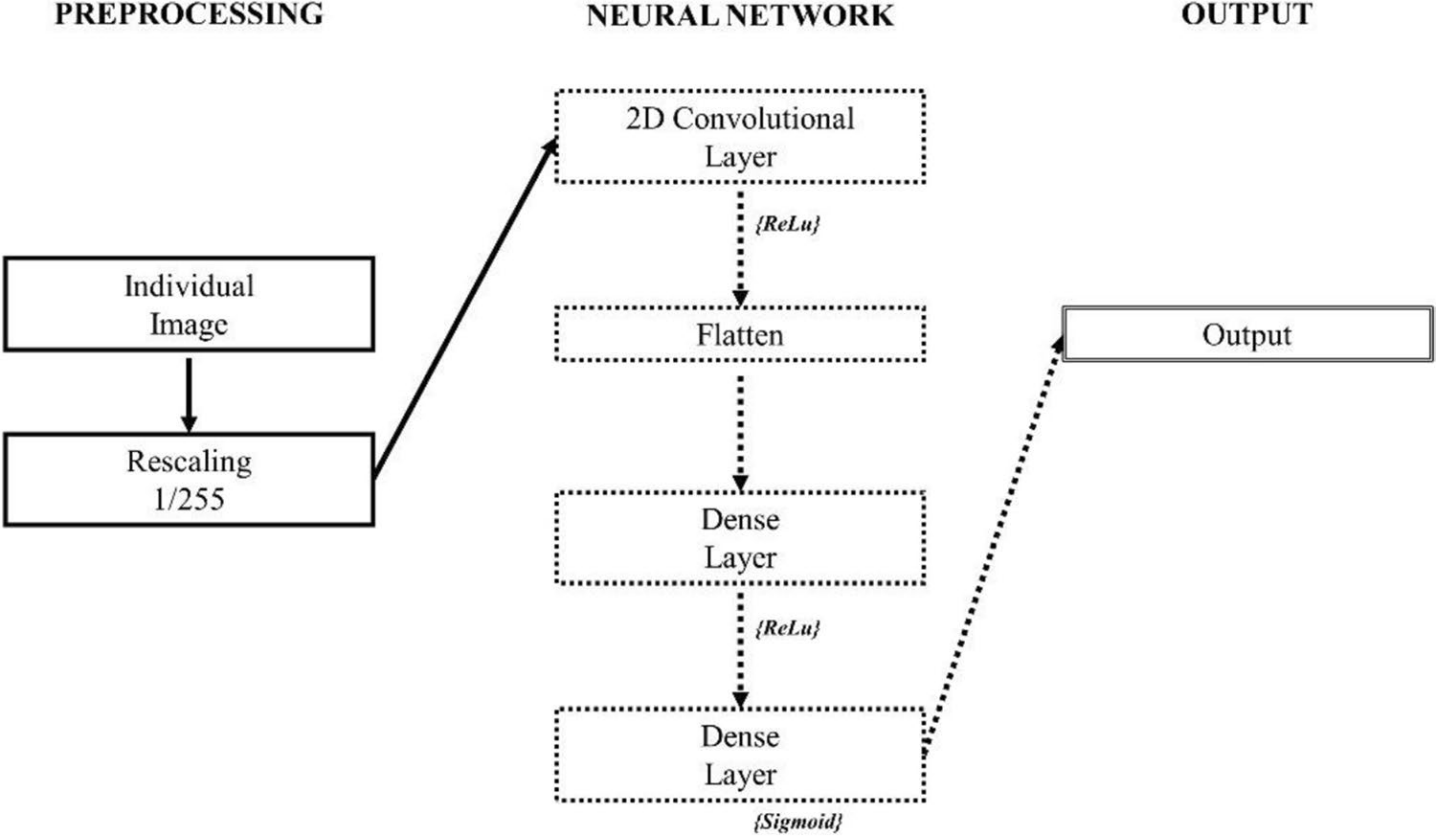
Flow of information through the Neural Network. ReLu = Rectified Linear Activation

A Convolutional Neural Network (CNN) was computed in order to discriminate between neurotypicals (TYP) and psychiatric participants. Each diagnostic group (participants with Schizophrenia – SCH; participants with a diagnosis of Bipolar Disorder – BIP; participants with a diagnosis of Attention Deficit/Hyperactivity Disorder – ADHD) was compared to neurotypicals, and the resulting Precision-Recall Area Under the Curve (PR-AUC) for the model was presented. The neural network was built using *Python 3.8.5* (Van Rossum & Drake, 2009) and the following libraries: *TensorFlow* (Martín Abadi et al., 2015), *Keras* (Chollet, 2015). The neural network had the following structure: firstly, images were scaled to low resolution (14*1 pixel, one band of color per ROI, each row for a different subject). The overall sample was divided in a train and test set through a 80/20 ratio. The first layer rescaled pixel RGB values from the [0,255] range to [0,1]. Preprocessed data then served as the input to a convolutional neural network with activation pattern *Rectified Linear Activation Function* and sliding window of size 1 on the x-axis, 3 on the y-axis. A flattening layer was then added, and two dense layers were built as the final steps. The first dense layer had 4 neurons, and activation pattern *Rectified Linear Activation Function.* The second dense layer had 1 final neuron with activation pattern S*igmoid Function*. The CNN models were built using optimizer *adam* (Kingma & Ba, 2017), loss was defined as the *binary cross-entropy* (Boer et al., 2005). A graphical representation of the flow of information, from input to output, of the neural net was offered in Fig. 1.

### Control analyses – baseline models and the role of motion

Logistic models (GLM) were built in order to evaluate the discriminative power through interpretable and more commonly used Machine Learning Algorithms. The GLM models were built for each diagnostic label compared to neurotypicals and served as a baseline model to compare and contrast CNN results. All the parameters used for building individual subject’s images were used for the prediction (ECM, fALFF, ReHo values for each ROI – for a total of 42 features). As for the CNN model, the overall sample was split in a 80/20 ratio between test and training sets. The discriminative power was evaluated through the PR-AUC on the test set.

As motion during scan is a common source of noise in fMRI (Makowski et al., 2019), the authors investigated baseline models constructed on mean Framewise Displacement (Power et al., 2014) values per scan per subject. As for the other analyses, the overall sample was split in 80/20 ratio between test and training sets. The discriminative power was evaluated through the PR-AUC on the test set.

GLM models were built in *R 4.0.3* (R Core Team, 2020), using *RStudio 1.3.1093* (RStudio Team, 2020) and using the following libraries: *tidyverse* (Wickham et al., 2019)*, caret* (Kuhn, 2008)*, PRROC* (Keilwagen et al., 2014).

## Results

### Primary results

**Fig. 2:**
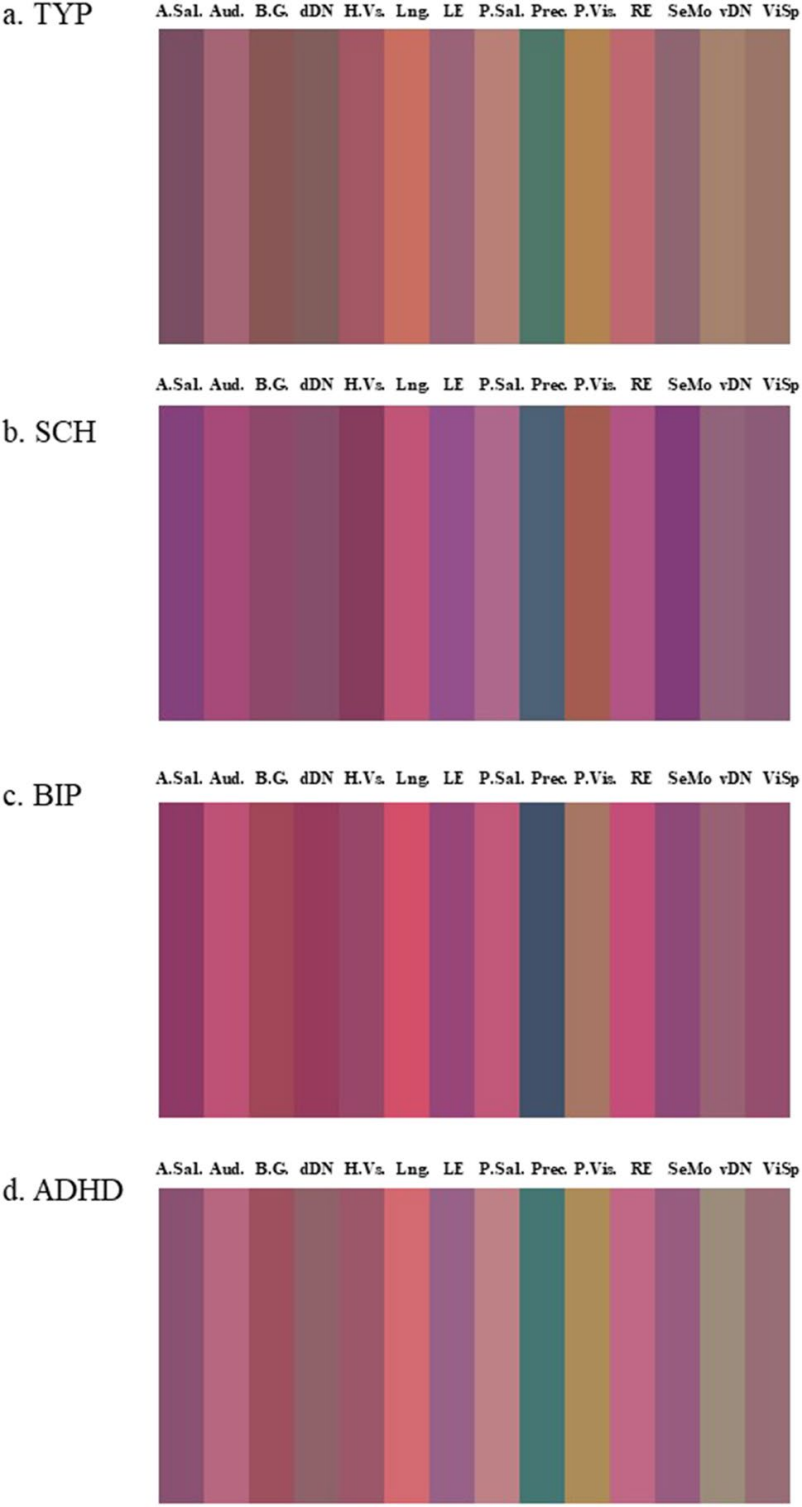
Average Image per diagnostic group. Images were obtained through an additive color method through RGB coding: Eigenvector Centrality for the red channel, fractional Amplitude of Low-Frequency Fluctuations for the green channel and Regional Homogeneity for the blue channel. A. Sal = Anterior Salience Aud. = Auditory Network B.G. = Basal Ganglia dDN = Dorsal Default Mode Network H.Vs. = Higher Visual Network Lng. = Language Network LE = Left Executive Control Network P.Sal = Posterior Salience Prec. = Precuneus P.Vis. = Primary Visual Network RE = Right Executive Control Network SeMo = Sensorimotor Cortex vDN = Ventral Default Mode Network ViSp = Visuospatial Network TYP = neurotypicals SCH = participants with Schizophrenia BIP = participants with Bipolar Disorder ADHD = participants with Attention Deficit/Hyperactivity Disorder

Average images per diagnostic group were plotted. Each image was composed by 14 bands of colors, one for each Functional Network. The resulting image was presented as Fig. 2. By a visual inspection, the average image for the participants with Schizophrenia had a higher component of purple color (+ Red -Green + Blue) in comparison to neurotypicals. The average image for the patients with Bipolar Disorder in comparison to neurotypicals had a higher component of purple color as well. The average image for the patients with ADHD showed similar color components than neurotypicals. Of particular interests, the Precuneus showed a prevalence of fALFF components (Green) in neurotypicals and ADHD participants, whereas a higher presence of ReHo components (Blue) in participants with Schizophrenia or Bipolar Disorder. The ECM component (Red) was low in the overall sample for the region.

**Fig. 3:**
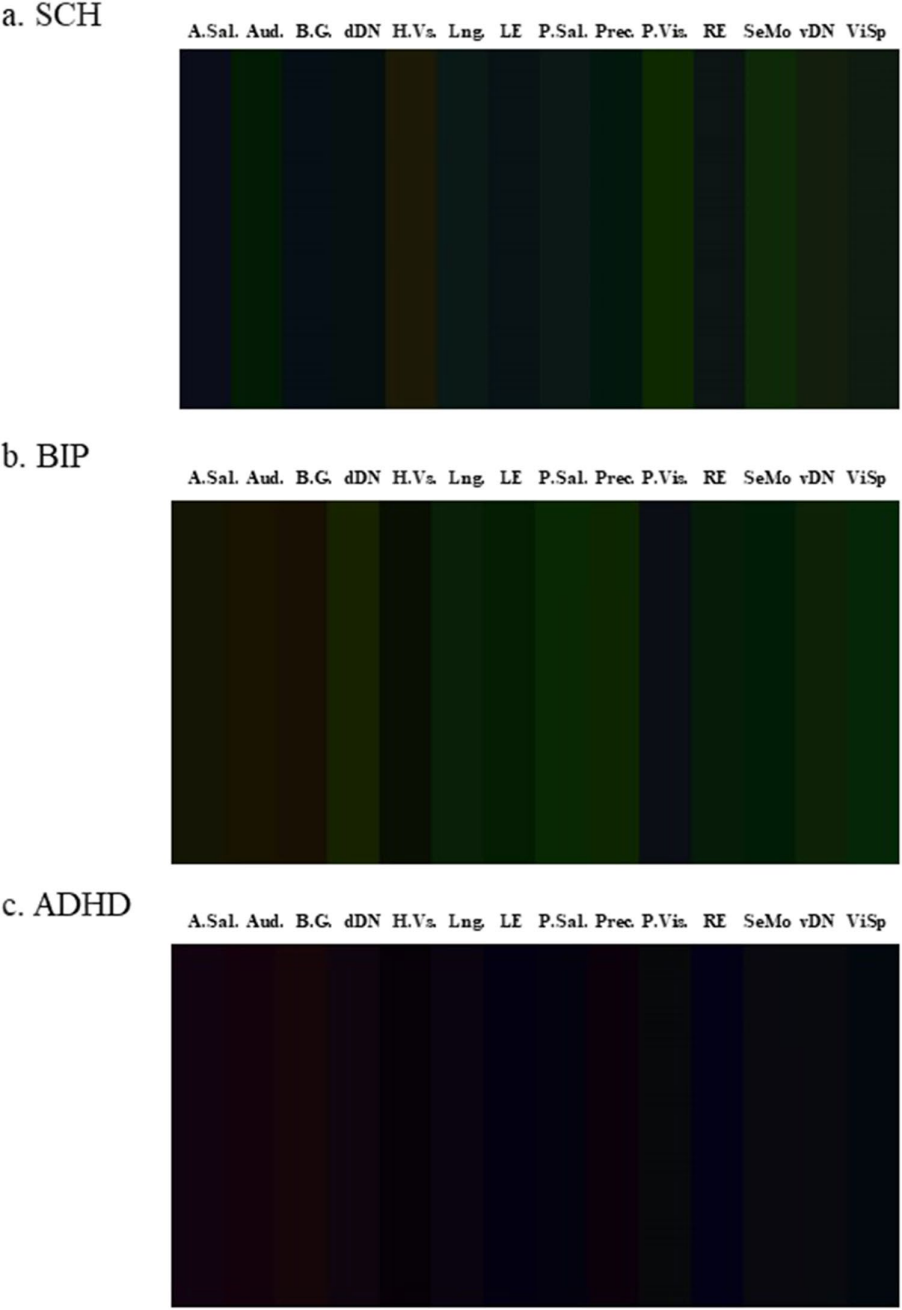
Average Image per diagnostic group, difference to neurotypicals. Images were obtained through an additive color method through RGB coding: Eigenvector Centrality for the red channel, fractional Amplitude of Low-Frequency Fluctuations for the green channel and Regional Homogeneity for the blue channel. A.Sal = Anterior Salience Aud. = Auditory Network B.G. = Basal Ganglia dDN = Dorsal Default Mode Network H.Vs. = Higher Visual Network Lng. = Language Network LE = Left Executive Control Network P.Sal = Posterior Salience Prec. = Precuneus P.Vis. = Primary Visual Network RE = Right Executive Control Network SeMo = Sensorimotor Cortex vDN = Ventral Default Mode Network ViSp = Visuospatial Network TYP = neurotypicals SCH = participants with Schizophrenia BIP = participants with Bipolar Disorder ADHD = participants with Attention Deficit/Hyperactivity Disorder

Subtracted images (diagnostic group – neurotypicals) were plotted as Fig. 3. The group of participants with Schizophrenia and Bipolar Disorder had similar prevalence of green components overall (fALFF higher in neurotypicals), but with visible differences in the dorsal and ventral Default Mode Networks, High Visual Network, Left and Right Executive Control Network, Primary Visual Network and the Visuospatial Network. The group of participants with Schizophrenia and Bipolar Disorder differed in the anterior Salience Network, Auditory Network and Basal Ganglia, with a higher prevalence of blue (ReHo) in Schizophrenia and a higher presence of red (ECM) in Bipolar Disorder. The sample of participants with ADHD had a prevalence of black (0 values, as the two groups had comparable mean values) and blue (ReHo, higher in ADHD).

**Fig. 4:**
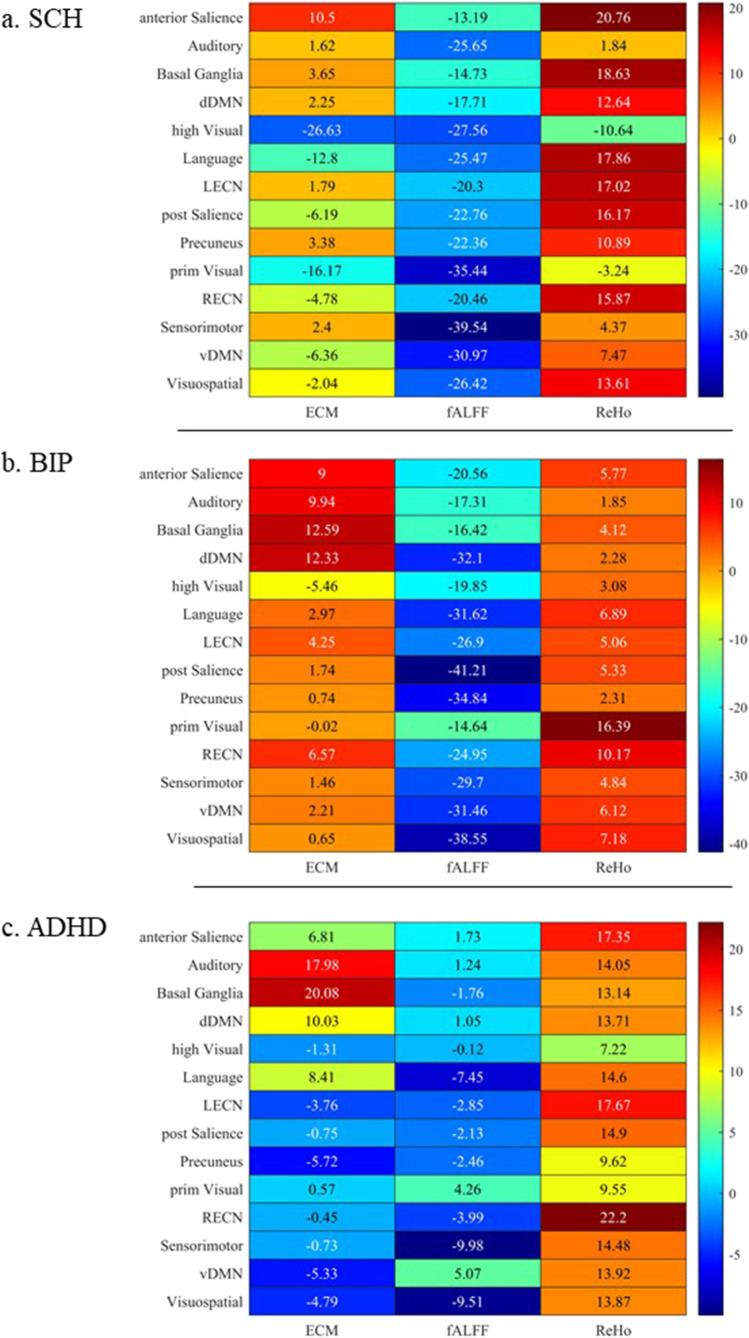
Heatmap representing differences to neurotypicals, per feature and ROI. TYP = neurotypicals SCH = participants with Schizophrenia BIP = participants with Bipolar DisorderADHD = participants with Attention Deficit/Hyperactivity Disorder

Numerical differences to neurotypicals were also plotted as a heatmap in Fig. 4.

MANOVA results were described in Supplementary Table S1, S2 and S3.

Only the group of participants with a diagnosis of Schizophrenia had significant differences for ECM results (p-value 0.045). Post-hoc ANOVA test were significant for the High Visual Network, Posterior Salience Network, Right Executive Control Network, ventral Default Mode Network, and the Visuospatial Network (minimum p-value 0.008, maximum 0.040). ECM results were reported in Supplementary Table S1.

For what concerned fALFF results, both the group of participants with a diagnosis of Schizophrenia and Bipolar Disorder had significant differences in comparison to neurotypicals (p-value 0.032 and < 0.001 respectively). Post-hoc ANOVA test were significant in the Auditory Network, Language Network, Precuneus, Sensorimotor Network, ventral Default Mode Network, Visuospatial Network for both the group of participants with a diagnosis of Schizophrenia and Bipolar Disorder (minimum p-value < 0.001, maximum 0.042). Participants with Schizophrenia also showed significant difference in comparison to neurotypicals in the High Visual Network and Primary Visual Network, while participants with Bipolar Disorder in the dorsal Default Mode Network, Anterior and Posterior Salience Network, Left and Right Executive Control Networks. fALFF results were reported in Supplementary Table S2.

ReHo showed significant differences for the Left Executive Network for both participants with Schizophrenia and ADHD, although borderline significant (p-value 0.040 and 0.050 respectively). The Posterior Salience also had borderline significant results for participants with Schizophrenia (p-value 0.049). Participants with Schizophrenia had statistically significant differences for ReHo in the Anterior Salience and Language Networks as well as the Basal Ganglia (p-values 0.015; 0.025 and 0.027 respectively). Participants with Bipolar Disorder exhibited significant differences for ReHo in the Primary Visual Network (p-value 0.011) and participants with ADHD in the Right Executive Control Network (p-value 0.017). ReHo results were reported in Supplementary Table S3.

### Secondary Results – discriminative power

One Convolutional Neural Network (CNN) per diagnostic group was built in order to discriminate between case (psychiatric participants) and controls (neurotypicals). The classification ability of the CNNs were evaluated through their Precision-Recall AUC (PR-AUC) on the test-set. All the models reached a PR-AUC > 80%. Results were described in Table 1. The highest PR-AUC was reached for the sample of patients suffering from Bipolar Disorder (96.8%), followed by the sample of patients suffering from Schizophrenia (91.8%) and patients with ADHD (84.6%).

**Table 1:** Discriminative Power, CNN and control analyses

Precision-Recall AUC: method	SCH	BIP	ADHD
CNN	91.8%	96.8%	84.6%
GLM integrated data	77.8%	68.5%	78.5%
GLM motion	60.8%	62.7%	65.9%

**Note**: Precision-Recall AUC measured on the validation sample (80/20 split)

Motion = mean Framewise Displacement values per subject per run

Control group: TYP

CNN = Convolutional Neural Network

GLM = Logistic Model

TYP = neurotypicals

SCH = participants with a diagnosis of Schizophrenia

BIP = participants with a diagnosis of Bipolar Disorder

ADHD = participants with a diagnosis of Attention Deficit/Hyperactivity Disorder

### Control analyses– baseline models and the role of motion

GLM models computed on the same data used to construct individual integrated images resulted in an overall lower discriminative power in comparison to CNN algorithms. PR-AUC was measured on the test-set. The highest PR-AUC was reached for the ADHD sample (78.5% GLM vs 84.62% CNN). Both the sample of participants with a diagnosis of Schizophrenia (77.8% GLM vs 91.8% CNN) and Bipolar Disorder (68.5% GLM vs 96.8% CNN) reported significantly lower PR-AUC values in comparison to CNN results. Results were reported in Table 1.

When GLM models were trained on mean FD values per subject per run, their predictive power for diagnostic label was evaluated. The discriminative power of GLM models evaluated on the test-set and built on motion parameters was moderate, but significantly lower than GLM models built on integrated data or CNN models. The highest PR-AUC was reached in the ADHD sample (65.9%), and the lowest in the sample of participants with a diagnosis of Schizophrenia (60.8%). The sample of participants with a diagnosis of Bipolar Disorder was moderate (62.7%). Results were reported in Table 1.

## Discussion

### Clinical significance and future prospects

In the development of the presented methodology, the authors focused on color-coding as a scheme of representing higher-order information through a simple and human readable content. Indeed, color schemes have long been used in communication technology as secondary notation. Color coding seems to offer a quick system of reference which may be easily discriminated by the human eye (Rozak & Rozak, 2014), aiding in reducing the perceived complexity of presented information (Yuditsky et al., 2002) as well as increasing consistency in the derived decision-making processes (Jonker et al., 2019). As recently stated in a review over neuroimaging tools for the psychiatric clinical practice (Scarpazza et al., 2020), most tools available to the present day were developed and validated for neurological disorders and are not suitable for application in the general psychiatric setting. Moreover, the authors suggested moving from a region-of-interest to a whole-brain approach, as well as accounting for disease heterogeneity (Scarpazza et al., 2020). In authors’ opinion, i-ECO well addresses both suggestions in an accessible manner.

While the current study supports the usage of i-ECO to classify fMRI participants according to diagnostic groups, considering previous discussed views offered by the clinical setting, the potential of a dimensional approach seems warranted. According to previous research in fact, neuroimaging biomarkers may have the potential to find different correspondences of psychopathology (Kebets et al., 2019; McTeague et al., 2017), in order to arrive to a more specific definition of cornerstone symptoms, their biological correlates and overall classifications supported by experimental results (Chang et al., 2020; Iravani et al., 2021; Schilbach et al., 2015; Tokuda et al., 2018). An integrated approach to neuroimaging has the potential for direct implications in the treatment of mental suffering and psychiatric practice (Iravani et al., 2021; Price et al., 2018), through a coordination of theoretical models for general psychiatry, psychotherapy, and neuroimaging—e.g. attachment theory and depression (X. Zhang et al., 2011); body image distortion and eating disorders (Castellini et al., 2013); face discrimination and gender incongruence (Fisher et al., 2020). The current study and its proposed novel methodology may thus aid clinicians in overcoming the technical barrier of entry to the field of neuroimaging for what concerns fMRI results. In addition to the psychiatric field, fMRI has been employed in the study of at-risk regions of the brain during the planning of neurosurgery (Unadkat et al., 2019). While early usage focused on functional mapping during task-based fMRI (Unadkat et al., 2019), recent developments integrated insight offered by resting-state information – either by functional connectivity, centrality measures or spectral dimensions (D’Andrea, Trillo’, Picotti, & Raco, 2017; Hart et al., 2016; Shimony et al., 2009). fMRI has also been investigated in the study of neurological disorders, in particular migraine (Schwedt et al., 2015) or neurodegenerative diseases (Rodriguez-Raecke et al., 2021), as well as other general clinical conditions (Chen et al., 2021; C. Liu et al., 2021). No formal rationale seems to arise in favor a limitation of i-ECO to the psychiatric field alone, as the proposed measures it relies upon seem to have a confirmed role over a wide array of clinical diagnoses. Therefore, the authors would like to invite clinical feedback to the project, in order to further enhance i-ECO as a methodology and extend its scope of applications.

The authors welcome clinical feedbacks to the project in order to further enhance the i-ECO methodology.

### Partial overlap schizophrenia and bipolar disorder

Our results supported an interpretation in favor of a global difference in fMRI components for the sample of patients suffering from either Schizophrenia or Bipolar Disorder in comparison to controls. In particular, a higher dominance of ECM and ReHo components was appreciable and confirmed by the secondary analyses. fALFF resulted less represented in the sample of patients suffering from either Schizophrenia or Bipolar Disorder in comparison to controls. These findings seem to be supported by recent studies in the field of Resting-State fMRI (Duan et al, 2017; C. Zhou et al., 2019a, b; Q. Zhou et al., 2017).

The partial overlap of findings between the sample of patients suffering from Schizophrenia and Bipolar Disorder can be interpreted in light of the clinical account of a continuity between the two disorders (Möller, 2003; Salagre et al., 2020). A continuity in the spectrum between Schizophrenia and Bipolar Disorder seems to be also supported by previous remarks of common and shared biomarkers (Yamada et al., 2020), from common genetic determinants (Lichtenstein et al., 2009; Prata et al., 2019) to shared neuroimaging features (Ji et al., 2019; Jimenez et al., 2019; Madre et al., 2020). This difficulty in differentiating between Schizophrenia and Bipolar Disorder is similar to the one that emerges in clinical practice when considering the diagnosis at a single time-point without the help of a longitudinal perspective (Rosen et al., 2011).

### ADHD specificity and role of precuneus

In contrast to the sample of patients suffering from either Schizophrenia or Bipolar Disorder, our results supported an interpretation of a local rather than global difference of fMRI components for the ADHD sample in comparison to neurotypical controls. Our findings suggested a potential role for the precuneus, which seems supported by previous literature (Castellanos et al., 2008; Christakou et al., 2013). Precuneus’ findings might be interpreted in light of its role in the default mode network (Cunningham et al., 2017; R. Li et al., 2019), and specifically in its suggested involvement in the activation/deactivation as part of the default mode network during task (Christakou et al., 2013). The role of precuneus in response inhibition seems to be of particular interest when considering the clinical presentation of ADHD, as the precuneus has been shown to be related to response inhibition (Albert et al., 2019), emotion regulation and attentional deployment (Ferri et al., 2016; B. Li et al., 2020). A role for the precuneus in ADHD seems to be supported also by reports of normalization in precuneus connectivity at the fMRI after methylphenidate or atomoxetine treatment (Kowalczyk et al., 2019), along symptomatic amelioration.

### Technical contributions

Dimensionality reduction represents one of the pivotal challenges in fMRI (Pereira et al., 2009), with most commonly used approaches – e.g. Principal Components Analysis, Autoregression, Linear Embeddings, Autoencoders (Cordes & Nandy, 2006; Huang et al., 2018; Mannfolk et al., 2010; Pereira et al., 2009) being of difficult interpretation for clinicians and hard to generalize. Research in fMRI has been recently characterized by a higher reliance on Neural Networks (Suk et al., 2016) and Embeddings (Sidhu, 2019), with the most promising results coming from CNN (Meszlényi et al., 2017; Sarraf et al., 2019; Tahmassebi et al., 2018; Zhao et al., 2018), especially in the field of Computational Psychiatry (Ariyarathne et al., 2020; El Gazzar et al., 2019; Oh et al., 2019; Silva et al., 2021). Convolutional Neural Networks design follows biological research and the study of the receptive field by the visual cortex (Hubel & Wiesel, 1959), their first development establishing the groundwork for the field of computer vision (Denker et al., 1989; LeCun et al., 1989). The present work establishes a direct connection between computer vision, fMRI data and CNN classifiers, providing a simple interpretation to the reason why CNN deep learning algorithms have been highly efficient when analyzing fMRI results. When fMRI data is recognized as a collection of spatially dispersed temporal components (either derived from functional connectivity, graph-network theory, or spectral analysis), and interpreted visually, the abstractness of a CNN classifier is reduced. Recent developments in the interpretation of variable importance for CNN models (Mijolla et al., 2020; Malmgren-Hansen et al., 2021) may shed further light over the contribution of the three fMRI components here analyzed, thus enriching the interpretation offered by the authors.

Although motion parameters seemed to have a moderate predictive power for psychiatric diagnoses, the overall precision of classifier models was highest when using an integrated approach to fMRI results. A convolutional network approach to classifiers seemed to increase the precision and recall in order to classify participants as either neurotypicals or suffering from a mental disorder. These preliminary results highlight the importance of motion in fMRI (Bolton et al., 2020; Makowski et al., 2019), but also caution authors to consider the information given by established measures in the field for the study and evaluation of psychiatric disorders.

### Limitations

The sample size in this study was limited and only comprised participants in a range of three different psychiatric diagnoses: Schizophrenia, Bipolar Disorder, ADHD. Further analyses with a multi-class.

approach are warranted before the generalization of its results. An evaluation of the discriminative power of this method over different diagnoses is warranted before generalizing its results on the overall general psychiatric field.

## Conclusions

In conclusion, this study provides preliminary evidence in support of an integrative approach in the analysis and visualization of fMRI results. The usage of macro-level regions, although diluting particular signals in specific brain areas, seemed to provide a high discriminative power for psychiatric disorders. This proof-of-work may serve to investigate further developments over more extensive dataset and over a different range of psychiatric diagnoses.

## Supplementary Information

Below is the link to the electronic supplementary material.

Supplementary file1 (DOCX 80 KB)

## Abbreviations

ADHD: Attention Deficit Hyperactivity Disorder
AFNI: Analysis of Functional NeuroImages
AUC: Area Under the Curve
BIP: Bipolar Disorder
BOLD: Blood Oxygenation Level Dependent
CNN: Convolutional Neural Network
ECM: Eigenvector Centrality
fALFF: Fractional Amplitude of Low-Frequency Fluctuation
FFT: Fast Fourier Transform
FDR: False Discovery Rate
fMRI: Functional Magnetic Resonance Imaging
KCC: Kendall’s Coefficient of Concordance
i-ECO: Integrated-Explainability through Color Coding
MEG: Magnetoencephalography
MENT: Participants with any Mental Disorder
MNI: Montreal Neurological Institute
ROI: Region/regions of interest
TYP: Neurotypicals
SCH: Participants with Schizophrenia

## Declarations

### Data availability

The data supporting the findings of this study are available from the corresponding author upon reasonable request.
