# Supplementary material for: The colors of our brain: an integrated approach for dimensionality reduction and explainability in fMRI through color coding (i-ECO)
Source: Brain Imaging Behav. 2021 Oct 24;16(3):977–90. doi: 10.1007/s11682-021-00584-8 (PMC9107439; doi:10.1007/s11682-021-00584-8)
Supplement: Supplementary file 1 — Supplementary file1 (DOCX 80 KB) [file 11682_2021_584_MOESM1_ESM.docx]

# SUPPLEMENTARY MATERIALS

| **Table S1a – MANOVA, ECM results**  **Pillai Test** | | | | | | | | | | | | | |
| --- | --- | --- | --- | --- | --- | --- | --- | --- | --- | --- | --- | --- | --- |
| **Cases** | | **df** | | **Approx. F** | | **Trace _Pillai_** | | **Num df** | | **Den df** | | **p** | |
| (Intercept) |  | 1 |  | 4035.319 |  | 0.997 |  | 14 |  | 185.000 |  | 1.119e -221 |  |
| SCH |  | 1 |  | 1.777 |  | 0.119 |  | 14 |  | 185.000 |  | 0.045 |  |
| BIP |  | 1 |  | 0.539 |  | 0.039 |  | 14 |  | 185.000 |  | 0.907 |  |
| ADHD |  | 1 |  | 0.291 |  | 0.022 |  | 14 |  | 185.000 |  | 0.994 |  |
| Residuals |  | 198 |  |  |  |  |  |  |  |  |  |  |  |
|  | | | | | | | | | | | | | |

| **Wilks Test** | | | | | | | | | | | | | |
| --- | --- | --- | --- | --- | --- | --- | --- | --- | --- | --- | --- | --- | --- |
| **Cases** | | **df** | | **Approx. F** | | **Wilks' Λ** | | **Num df** | | **Den df** | | **p** | |
| (Intercept) |  | 1 |  | 4035.319 |  | 0.003 |  | 14 |  | 185.000 |  | 1.119e -221 |  |
| SCH |  | 1 |  | 1.777 |  | 0.881 |  | 14 |  | 185.000 |  | 0.045 |  |
| BIP |  | 1 |  | 0.539 |  | 0.961 |  | 14 |  | 185.000 |  | 0.907 |  |
| ADHD |  | 1 |  | 0.291 |  | 0.978 |  | 14 |  | 185.000 |  | 0.994 |  |
| Residuals |  | 198 |  |  |  |  |  |  |  |  |  |  |  |
|  | | | | | | | | | | | | | |

| **Hotelling-Lawley Test** | | | | | | | | | | | | | |
| --- | --- | --- | --- | --- | --- | --- | --- | --- | --- | --- | --- | --- | --- |
| **Cases** | | **df** | | **Approx. F** | | **Trace _H-L_** | | **Num df** | | **Den df** | | **p** | |
| (Intercept) |  | 1 |  | 4035.319 |  | 305.376 |  | 14 |  | 185.000 |  | 1.119e -221 |  |
| SCH |  | 1 |  | 1.777 |  | 0.134 |  | 14 |  | 185.000 |  | 0.045 |  |
| BIP |  | 1 |  | 0.539 |  | 0.041 |  | 14 |  | 185.000 |  | 0.907 |  |
| ADHD |  | 1 |  | 0.291 |  | 0.022 |  | 14 |  | 185.000 |  | 0.994 |  |
| Residuals |  | 198 |  |  |  |  |  |  |  |  |  |  |  |
|  | | | | | | | | | | | | | |

| **Roy Test** | | | | | | | | | | | | | |
| --- | --- | --- | --- | --- | --- | --- | --- | --- | --- | --- | --- | --- | --- |
| **Cases** | | **df** | | **Approx. F** | | **Largest Root** | | **Num df** | | **Den df** | | **p** | |
| (Intercept) |  | 1 |  | 4035.319 |  | 305.376 |  | 14 |  | 185.000 |  | 1.119e -221 |  |
| SCH |  | 1 |  | 1.777 |  | 0.134 |  | 14 |  | 185.000 |  | 0.045 |  |
| BIP |  | 1 |  | 0.539 |  | 0.041 |  | 14 |  | 185.000 |  | 0.907 |  |
| ADHD |  | 1 |  | 0.291 |  | 0.022 |  | 14 |  | 185.000 |  | 0.994 |  |
| Residuals |  | 198 |  |  |  |  |  |  |  |  |  |  |  |
|  | | | | | | | | | | | | | |

| **Table S1b – post-hoc ANOVA results, significant results for ECM  high_Visual_ECM** | | | | | | | | | | | |
| --- | --- | --- | --- | --- | --- | --- | --- | --- | --- | --- | --- |
| **Cases** | | **Sum of Squares** | | **df** | | **Mean Square** | | **F** | | **p** | |
| (Intercept) |  | 4.909e +6 |  | 1 |  | 4.909e +6 |  | 2073.765 |  | 7.287e -107 |  |
| SCH |  | 17218.792 |  | 1 |  | 17218.792 |  | 7.273 |  | 0.008 |  |
| BIP |  | 1844.781 |  | 1 |  | 1844.781 |  | 0.779 |  | 0.378 |  |
| ADHD |  | 778.197 |  | 1 |  | 778.197 |  | 0.329 |  | 0.567 |  |
| Residuals |  | 468748.575 |  | 198 |  | 2367.417 |  |  |  |  |  |
|  | | | | | | | | | | | |

| **post_Salience_ECM** | | | | | | | | | | | |
| --- | --- | --- | --- | --- | --- | --- | --- | --- | --- | --- | --- |
| **Cases** | | **Sum of Squares** | | **df** | | **Mean Square** | | **F** | | **p** | |
| (Intercept) |  | 6.959e +6 |  | 1 |  | 6.959e +6 |  | 4951.908 |  | 4.605e -142 |  |
| SCH |  | 6394.334 |  | 1 |  | 6394.334 |  | 4.550 |  | 0.034 |  |
| BIP |  | 1270.531 |  | 1 |  | 1270.531 |  | 0.904 |  | 0.343 |  |
| ADHD |  | 631.327 |  | 1 |  | 631.327 |  | 0.449 |  | 0.503 |  |
| Residuals |  | 278262.896 |  | 198 |  | 1405.368 |  |  |  |  |  |
|  | | | | | | | | | | | |

| **RECN_ECM** | | | | | | | | | | | |
| --- | --- | --- | --- | --- | --- | --- | --- | --- | --- | --- | --- |
| **Cases** | | **Sum of Squares** | | **df** | | **Mean Square** | | **F** | | **p** | |
| (Intercept) |  | 7.304e +6 |  | 1 |  | 7.304e +6 |  | 5651.401 |  | 1.536e -147 |  |
| SCH |  | 6140.741 |  | 1 |  | 6140.741 |  | 4.751 |  | 0.030 |  |
| BIP |  | 730.912 |  | 1 |  | 730.912 |  | 0.566 |  | 0.453 |  |
| ADHD |  | 189.440 |  | 1 |  | 189.440 |  | 0.147 |  | 0.702 |  |
| Residuals |  | 255903.774 |  | 198 |  | 1292.443 |  |  |  |  |  |
|  | | | | | | | | | | | |

| **Sensorimotor_ECM** | | | | | | | | | | | |
| --- | --- | --- | --- | --- | --- | --- | --- | --- | --- | --- | --- |
| **Cases** | | **Sum of Squares** | | **df** | | **Mean Square** | | **F** | | **p** | |
| (Intercept) |  | 4.093e +6 |  | 1 |  | 4.093e +6 |  | 1921.369 |  | 7.072e -104 |  |
| SCH |  | 5986.448 |  | 1 |  | 5986.448 |  | 2.810 |  | 0.095 |  |
| BIP |  | 121.082 |  | 1 |  | 121.082 |  | 0.057 |  | 0.812 |  |
| ADHD |  | 2069.756 |  | 1 |  | 2069.756 |  | 0.972 |  | 0.325 |  |
| Residuals |  | 421774.063 |  | 198 |  | 2130.172 |  |  |  |  |  |
|  | | | | | | | | | | | |

| **vDMN_ECM** | | | | | | | | | | | |
| --- | --- | --- | --- | --- | --- | --- | --- | --- | --- | --- | --- |
| **Cases** | | **Sum of Squares** | | **df** | | **Mean Square** | | **F** | | **p** | |
| (Intercept) |  | 5.088e +6 |  | 1 |  | 5.088e +6 |  | 3134.467 |  | 2.411e -123 |  |
| SCH |  | 6939.410 |  | 1 |  | 6939.410 |  | 4.275 |  | 0.040 |  |
| BIP |  | 2500.869 |  | 1 |  | 2500.869 |  | 1.541 |  | 0.216 |  |
| ADHD |  | 2245.576 |  | 1 |  | 2245.576 |  | 1.383 |  | 0.241 |  |
| Residuals |  | 321377.913 |  | 198 |  | 1623.121 |  |  |  |  |  |
|  | | | | | | | | | | | |

| **Visuospatial_ECM** | | | | | | | | | | | |
| --- | --- | --- | --- | --- | --- | --- | --- | --- | --- | --- | --- |
| **Cases** | | **Sum of Squares** | | **df** | | **Mean Square** | | **F** | | **p** | |
| (Intercept) |  | 4.646e +6 |  | 1 |  | 4.646e +6 |  | 5567.992 |  | 6.366e -147 |  |
| SCH |  | 5405.453 |  | 1 |  | 5405.453 |  | 6.478 |  | 0.012 |  |
| BIP |  | 563.930 |  | 1 |  | 563.930 |  | 0.676 |  | 0.412 |  |
| ADHD |  | 261.972 |  | 1 |  | 261.972 |  | 0.314 |  | 0.576 |  |
| Residuals |  | 165206.702 |  | 198 |  | 834.377 |  |  |  |  |  |
|  | | | | | | | | | | | |

**Note**: only ROIs with at least 1 significant result were reported

| **Table S2a – MANOVA, fALFF results Pillai Test** | | | | | | | | | | | | | |
| --- | --- | --- | --- | --- | --- | --- | --- | --- | --- | --- | --- | --- | --- |
| **Cases** | | **df** | | **Approx. F** | | **Trace _Pillai_** | | **Num df** | | **Den df** | | **p** | |
| (Intercept) |  | 1 |  | 172.870 |  | 0.929 |  | 14 |  | 185.000 |  | 3.930e -98 |  |
| SCH |  | 1 |  | 1.870 |  | 0.124 |  | 14 |  | 185.000 |  | 0.032 |  |
| BIP |  | 1 |  | 3.196 |  | 0.195 |  | 14 |  | 185.000 |  | 1.573e  -4 |  |
| ADHD |  | 1 |  | 1.045 |  | 0.073 |  | 14 |  | 185.000 |  | 0.411 |  |
| Residuals |  | 198 |  |  |  |  |  |  |  |  |  |  |  |
|  | | | | | | | | | | | | | |

| **Wilks Test** | | | | | | | | | | | | | |
| --- | --- | --- | --- | --- | --- | --- | --- | --- | --- | --- | --- | --- | --- |
| **Cases** | | **df** | | **Approx. F** | | **Wilks' Λ** | | **Num df** | | **Den df** | | **p** | |
| (Intercept) |  | 1 |  | 172.870 |  | 0.071 |  | 14 |  | 185.000 |  | 3.930e -98 |  |
| SCH |  | 1 |  | 1.870 |  | 0.876 |  | 14 |  | 185.000 |  | 0.032 |  |
| BIP |  | 1 |  | 3.196 |  | 0.805 |  | 14 |  | 185.000 |  | 1.573e  -4 |  |
| ADHD |  | 1 |  | 1.045 |  | 0.927 |  | 14 |  | 185.000 |  | 0.411 |  |
| Residuals |  | 198 |  |  |  |  |  |  |  |  |  |  |  |
|  | | | | | | | | | | | | | |

| **Hotelling-Lawley Test** | | | | | | | | | | | | | |
| --- | --- | --- | --- | --- | --- | --- | --- | --- | --- | --- | --- | --- | --- |
| **Cases** | | **df** | | **Approx. F** | | **Trace _H-L_** | | **Num df** | | **Den df** | | **p** | |
| (Intercept) |  | 1 |  | 172.870 |  | 13.082 |  | 14 |  | 185.000 |  | 3.930e -98 |  |
| SCH |  | 1 |  | 1.870 |  | 0.142 |  | 14 |  | 185.000 |  | 0.032 |  |
| BIP |  | 1 |  | 3.196 |  | 0.242 |  | 14 |  | 185.000 |  | 1.573e  -4 |  |
| ADHD |  | 1 |  | 1.045 |  | 0.079 |  | 14 |  | 185.000 |  | 0.411 |  |
| Residuals |  | 198 |  |  |  |  |  |  |  |  |  |  |  |
|  | | | | | | | | | | | | | |

| **Roy Test** | | | | | | | | | | | | | |
| --- | --- | --- | --- | --- | --- | --- | --- | --- | --- | --- | --- | --- | --- |
| **Cases** | | **df** | | **Approx. F** | | **Largest Root** | | **Num df** | | **Den df** | | **p** | |
| (Intercept) |  | 1 |  | 172.870 |  | 13.082 |  | 14 |  | 185.000 |  | 3.930e -98 |  |
| SCH |  | 1 |  | 1.870 |  | 0.142 |  | 14 |  | 185.000 |  | 0.032 |  |
| BIP |  | 1 |  | 3.196 |  | 0.242 |  | 14 |  | 185.000 |  | 1.573e  -4 |  |
| ADHD |  | 1 |  | 1.045 |  | 0.079 |  | 14 |  | 185.000 |  | 0.411 |  |
| Residuals |  | 198 |  |  |  |  |  |  |  |  |  |  |  |
|  | | | | | | | | | | | | | |

| **Table S2b – post-hoc ANOVA results, significant results for fALFF anterior_Salience_fALFF** | | | | | | | | | | | |
| --- | --- | --- | --- | --- | --- | --- | --- | --- | --- | --- | --- |
| **Cases** | | **Sum of Squares** | | **df** | | **Mean Square** | | **F** | | **p** | |
| (Intercept) |  | 1.082e +6 |  | 1 |  | 1.082e +6 |  | 1029.945 |  | 2.148e -80 |  |
| SCH |  | 2692.779 |  | 1 |  | 2692.779 |  | 2.564 |  | 0.111 |  |
| BIP |  | 12513.184 |  | 1 |  | 12513.184 |  | 11.916 |  | 6.805e  -4 |  |
| ADHD |  | 261.821 |  | 1 |  | 261.821 |  | 0.249 |  | 0.618 |  |
| Residuals |  | 207929.271 |  | 198 |  | 1050.148 |  |  |  |  |  |
|  | | | | | | | | | | | |

| **Auditory_fALFF** | | | | | | | | | | | |
| --- | --- | --- | --- | --- | --- | --- | --- | --- | --- | --- | --- |
| **Cases** | | **Sum of Squares** | | **df** | | **Mean Square** | | **F** | | **p** | |
| (Intercept) |  | 1.781e +6 |  | 1 |  | 1.781e +6 |  | 1007.466 |  | 1.340e -79 |  |
| SCH |  | 16399.587 |  | 1 |  | 16399.587 |  | 9.275 |  | 0.003 |  |
| BIP |  | 9513.898 |  | 1 |  | 9513.898 |  | 5.381 |  | 0.021 |  |
| ADHD |  | 113.333 |  | 1 |  | 113.333 |  | 0.064 |  | 0.800 |  |
| Residuals |  | 350084.831 |  | 198 |  | 1768.105 |  |  |  |  |  |
|  | | | | | | | | | | | |

| **Basal_Ganglia_fALFF** | | | | | | | | | | | |
| --- | --- | --- | --- | --- | --- | --- | --- | --- | --- | --- | --- |
| **Cases** | | **Sum of Squares** | | **df** | | **Mean Square** | | **F** | | **p** | |
| (Intercept) |  | 1.305e +6 |  | 1 |  | 1.305e +6 |  | 692.778 |  | 1.412e -66 |  |
| SCH |  | 2480.441 |  | 1 |  | 2480.441 |  | 1.317 |  | 0.252 |  |
| BIP |  | 5645.156 |  | 1 |  | 5645.156 |  | 2.998 |  | 0.085 |  |
| ADHD |  | 1014.968 |  | 1 |  | 1014.968 |  | 0.539 |  | 0.464 |  |
| Residuals |  | 372834.722 |  | 198 |  | 1883.004 |  |  |  |  |  |
|  | | | | | | | | | | | |

| **dDMN_fALFF** | | | | | | | | | | | |
| --- | --- | --- | --- | --- | --- | --- | --- | --- | --- | --- | --- |
| **Cases** | | **Sum of Squares** | | **df** | | **Mean Square** | | **F** | | **p** | |
| (Intercept) |  | 1.486e +6 |  | 1 |  | 1.486e +6 |  | 1039.637 |  | 9.854e -81 |  |
| SCH |  | 2517.154 |  | 1 |  | 2517.154 |  | 1.761 |  | 0.186 |  |
| BIP |  | 34052.448 |  | 1 |  | 34052.448 |  | 23.828 |  | 2.164e  -6 |  |
| ADHD |  | 614.607 |  | 1 |  | 614.607 |  | 0.430 |  | 0.513 |  |
| Residuals |  | 282965.035 |  | 198 |  | 1429.116 |  |  |  |  |  |
|  | | | | | | | | | | | |

| **high_Visual_fALFF** | | | | | | | | | | | |
| --- | --- | --- | --- | --- | --- | --- | --- | --- | --- | --- | --- |
| **Cases** | | **Sum of Squares** | | **df** | | **Mean Square** | | **F** | | **p** | |
| (Intercept) |  | 1.286e +6 |  | 1 |  | 1.286e +6 |  | 712.024 |  | 1.696e -67 |  |
| SCH |  | 16184.453 |  | 1 |  | 16184.453 |  | 8.960 |  | 0.003 |  |
| BIP |  | 6336.625 |  | 1 |  | 6336.625 |  | 3.508 |  | 0.063 |  |
| ADHD |  | 35.791 |  | 1 |  | 35.791 |  | 0.020 |  | 0.888 |  |
| Residuals |  | 357640.131 |  | 198 |  | 1806.263 |  |  |  |  |  |
|  | | | | | | | | | | | |

| **Language_fALFF** | | | | | | | | | | | |
| --- | --- | --- | --- | --- | --- | --- | --- | --- | --- | --- | --- |
| **Cases** | | **Sum of Squares** | | **df** | | **Mean Square** | | **F** | | **p** | |
| (Intercept) |  | 2.025e +6 |  | 1 |  | 2.025e +6 |  | 895.345 |  | 2.132e -75 |  |
| SCH |  | 9481.018 |  | 1 |  | 9481.018 |  | 4.192 |  | 0.042 |  |
| BIP |  | 23849.135 |  | 1 |  | 23849.135 |  | 10.545 |  | 0.001 |  |
| ADHD |  | 354.789 |  | 1 |  | 354.789 |  | 0.157 |  | 0.692 |  |
| Residuals |  | 447807.162 |  | 198 |  | 2261.652 |  |  |  |  |  |
|  | | | | | | | | | | | |

| **LECN_fALFF** | | | | | | | | | | | |
| --- | --- | --- | --- | --- | --- | --- | --- | --- | --- | --- | --- |
| **Cases** | | **Sum of Squares** | | **df** | | **Mean Square** | | **F** | | **p** | |
| (Intercept) |  | 1.681e +6 |  | 1 |  | 1.681e +6 |  | 1025.181 |  | 3.157e -80 |  |
| SCH |  | 5255.513 |  | 1 |  | 5255.513 |  | 3.205 |  | 0.075 |  |
| BIP |  | 21228.585 |  | 1 |  | 21228.585 |  | 12.948 |  | 4.046e  -4 |  |
| ADHD |  | 44.674 |  | 1 |  | 44.674 |  | 0.027 |  | 0.869 |  |
| Residuals |  | 324630.152 |  | 198 |  | 1639.546 |  |  |  |  |  |
|  | | | | | | | | | | | |

| **post_Salience_fALFF** | | | | | | | | | | | |
| --- | --- | --- | --- | --- | --- | --- | --- | --- | --- | --- | --- |
| **Cases** | | **Sum of Squares** | | **df** | | **Mean Square** | | **F** | | **p** | |
| (Intercept) |  | 2.825e +6 |  | 1 |  | 2.825e +6 |  | 1204.486 |  | 4.099e -86 |  |
| SCH |  | 7730.158 |  | 1 |  | 7730.158 |  | 3.296 |  | 0.071 |  |
| BIP |  | 44284.984 |  | 1 |  | 44284.984 |  | 18.881 |  | 2.222e  -5 |  |
| ADHD |  | 52.220 |  | 1 |  | 52.220 |  | 0.022 |  | 0.882 |  |
| Residuals |  | 464409.382 |  | 198 |  | 2345.502 |  |  |  |  |  |
|  | | | | | | | | | | | |

| **Precuneus_fALFF** | | | | | | | | | | | |
| --- | --- | --- | --- | --- | --- | --- | --- | --- | --- | --- | --- |
| **Cases** | | **Sum of Squares** | | **df** | | **Mean Square** | | **F** | | **p** | |
| (Intercept) |  | 2.405e +6 |  | 1 |  | 2.405e +6 |  | 1624.934 |  | 2.144e -97 |  |
| SCH |  | 6345.428 |  | 1 |  | 6345.428 |  | 4.287 |  | 0.040 |  |
| BIP |  | 37795.048 |  | 1 |  | 37795.048 |  | 25.534 |  | 9.854e  -7 |  |
| ADHD |  | 3.908 |  | 1 |  | 3.908 |  | 0.003 |  | 0.959 |  |
| Residuals |  | 293080.858 |  | 198 |  | 1480.206 |  |  |  |  |  |
|  | | | | | | | | | | | |

| **prim_Visual_fALFF** | | | | | | | | | | | |
| --- | --- | --- | --- | --- | --- | --- | --- | --- | --- | --- | --- |
| **Cases** | | **Sum of Squares** | | **df** | | **Mean Square** | | **F** | | **p** | |
| (Intercept) |  | 3.134e +6 |  | 1 |  | 3.134e +6 |  | 1228.706 |  | 7.514e -87 |  |
| SCH |  | 42642.106 |  | 1 |  | 42642.106 |  | 16.716 |  | 6.308e  -5 |  |
| BIP |  | 6494.933 |  | 1 |  | 6494.933 |  | 2.546 |  | 0.112 |  |
| ADHD |  | 2024.981 |  | 1 |  | 2024.981 |  | 0.794 |  | 0.374 |  |
| Residuals |  | 505085.649 |  | 198 |  | 2550.938 |  |  |  |  |  |
|  | | | | | | | | | | | |

| **RECN_fALFF** | | | | | | | | | | | |
| --- | --- | --- | --- | --- | --- | --- | --- | --- | --- | --- | --- |
| **Cases** | | **Sum of Squares** | | **df** | | **Mean Square** | | **F** | | **p** | |
| (Intercept) |  | 1.916e +6 |  | 1 |  | 1.916e +6 |  | 943.290 |  | 3.031e -77 |  |
| SCH |  | 5640.185 |  | 1 |  | 5640.185 |  | 2.777 |  | 0.097 |  |
| BIP |  | 17822.928 |  | 1 |  | 17822.928 |  | 8.774 |  | 0.003 |  |
| ADHD |  | 31.300 |  | 1 |  | 31.300 |  | 0.015 |  | 0.901 |  |
| Residuals |  | 402189.090 |  | 198 |  | 2031.258 |  |  |  |  |  |
|  | | | | | | | | | | | |

| **Sensorimotor_fALFF** | | | | | | | | | | | |
| --- | --- | --- | --- | --- | --- | --- | --- | --- | --- | --- | --- |
| **Cases** | | **Sum of Squares** | | **df** | | **Mean Square** | | **F** | | **p** | |
| (Intercept) |  | 1.581e +6 |  | 1 |  | 1.581e +6 |  | 731.986 |  | 1.973e -68 |  |
| SCH |  | 33265.874 |  | 1 |  | 33265.874 |  | 15.403 |  | 1.198e  -4 |  |
| BIP |  | 18509.716 |  | 1 |  | 18509.716 |  | 8.570 |  | 0.004 |  |
| ADHD |  | 2226.957 |  | 1 |  | 2226.957 |  | 1.031 |  | 0.311 |  |
| Residuals |  | 427632.323 |  | 198 |  | 2159.759 |  |  |  |  |  |
|  | | | | | | | | | | | |

| **vDMN_fALFF** | | | | | | | | | | | |
| --- | --- | --- | --- | --- | --- | --- | --- | --- | --- | --- | --- |
| **Cases** | | **Sum of Squares** | | **df** | | **Mean Square** | | **F** | | **p** | |
| (Intercept) |  | 2.999e +6 |  | 1 |  | 2.999e +6 |  | 1522.943 |  | 6.431e -95 |  |
| SCH |  | 18558.612 |  | 1 |  | 18558.612 |  | 9.425 |  | 0.002 |  |
| BIP |  | 33322.223 |  | 1 |  | 33322.223 |  | 16.923 |  | 5.707e  -5 |  |
| ADHD |  | 2163.683 |  | 1 |  | 2163.683 |  | 1.099 |  | 0.296 |  |
| Residuals |  | 389878.594 |  | 198 |  | 1969.084 |  |  |  |  |  |
|  | | | | | | | | | | | |

| **Visuospatial_fALFF** | | | | | | | | | | | |
| --- | --- | --- | --- | --- | --- | --- | --- | --- | --- | --- | --- |
| **Cases** | | **Sum of Squares** | | **df** | | **Mean Square** | | **F** | | **p** | |
| (Intercept) |  | 2.239e +6 |  | 1 |  | 2.239e +6 |  | 1078.504 |  | 4.602e -82 |  |
| SCH |  | 8189.954 |  | 1 |  | 8189.954 |  | 3.946 |  | 0.048 |  |
| BIP |  | 36049.878 |  | 1 |  | 36049.878 |  | 17.368 |  | 4.599e  -5 |  |
| ADHD |  | 1591.913 |  | 1 |  | 1591.913 |  | 0.767 |  | 0.382 |  |
| Residuals |  | 410975.084 |  | 198 |  | 2075.632 |  |  |  |  |  |
|  | | | | | | | | | | | |

**Note**: only ROIs with at least 1 significant result were reported

| **Table 3a – MANOVA, ReHo results Pillai Test** | | | | | | | | | | | | | |
| --- | --- | --- | --- | --- | --- | --- | --- | --- | --- | --- | --- | --- | --- |
| **Cases** | | **df** | | **Approx. F** | | **Trace _Pillai_** | | **Num df** | | **Den df** | | **p** | |
| (Intercept) |  | 1 |  | 160.659 |  | 0.924 |  | 14 |  | 185.000 |  | 2.029e -95 |  |
| SCH |  | 1 |  | 4.166 |  | 0.240 |  | 14 |  | 185.000 |  | 2.514e  -6 |  |
| BIP |  | 1 |  | 1.406 |  | 0.096 |  | 14 |  | 185.000 |  | 0.154 |  |
| ADHD |  | 1 |  | 0.846 |  | 0.060 |  | 14 |  | 185.000 |  | 0.619 |  |
| Residuals |  | 198 |  |  |  |  |  |  |  |  |  |  |  |
|  | | | | | | | | | | | | | |

| **Wilks Test** | | | | | | | | | | | | | |
| --- | --- | --- | --- | --- | --- | --- | --- | --- | --- | --- | --- | --- | --- |
| **Cases** | | **df** | | **Approx. F** | | **Wilks' Λ** | | **Num df** | | **Den df** | | **p** | |
| (Intercept) |  | 1 |  | 160.659 |  | 0.076 |  | 14 |  | 185.000 |  | 2.029e -95 |  |
| SCH |  | 1 |  | 4.166 |  | 0.760 |  | 14 |  | 185.000 |  | 2.514e  -6 |  |
| BIP |  | 1 |  | 1.406 |  | 0.904 |  | 14 |  | 185.000 |  | 0.154 |  |
| ADHD |  | 1 |  | 0.846 |  | 0.940 |  | 14 |  | 185.000 |  | 0.619 |  |
| Residuals |  | 198 |  |  |  |  |  |  |  |  |  |  |  |
|  | | | | | | | | | | | | | |

| **Hotelling-Lawley Test** | | | | | | | | | | | | | |
| --- | --- | --- | --- | --- | --- | --- | --- | --- | --- | --- | --- | --- | --- |
| **Cases** | | **df** | | **Approx. F** | | **Trace _H-L_** | | **Num df** | | **Den df** | | **p** | |
| (Intercept) |  | 1 |  | 160.659 |  | 12.158 |  | 14 |  | 185.000 |  | 2.029e -95 |  |
| SCH |  | 1 |  | 4.166 |  | 0.315 |  | 14 |  | 185.000 |  | 2.514e  -6 |  |
| BIP |  | 1 |  | 1.406 |  | 0.106 |  | 14 |  | 185.000 |  | 0.154 |  |
| ADHD |  | 1 |  | 0.846 |  | 0.064 |  | 14 |  | 185.000 |  | 0.619 |  |
| Residuals |  | 198 |  |  |  |  |  |  |  |  |  |  |  |
|  | | | | | | | | | | | | | |

| **Roy Test** | | | | | | | | | | | | | |
| --- | --- | --- | --- | --- | --- | --- | --- | --- | --- | --- | --- | --- | --- |
| **Cases** | | **df** | | **Approx. F** | | **Largest Root** | | **Num df** | | **Den df** | | **p** | |
| (Intercept) |  | 1 |  | 160.659 |  | 12.158 |  | 14 |  | 185.000 |  | 2.029e -95 |  |
| SCH |  | 1 |  | 4.166 |  | 0.315 |  | 14 |  | 185.000 |  | 2.514e  -6 |  |
| BIP |  | 1 |  | 1.406 |  | 0.106 |  | 14 |  | 185.000 |  | 0.154 |  |
| ADHD |  | 1 |  | 0.846 |  | 0.064 |  | 14 |  | 185.000 |  | 0.619 |  |
| Residuals |  | 198 |  |  |  |  |  |  |  |  |  |  |  |
|  | | | | | | | | | | | | | |

| **Table 3b – post-hoc ANOVA results, significant results for ReHo**  **anterior_Salience_ReHo** | | | | | | | | | | | |
| --- | --- | --- | --- | --- | --- | --- | --- | --- | --- | --- | --- |
| **Cases** | | **Sum of Squares** | | **df** | | **Mean Square** | | **F** | | **p** | |
| (Intercept) |  | 2.286e +6 |  | 1 |  | 2.286e +6 |  | 1011.849 |  | 9.352e -80 |  |
| SCH |  | 13508.789 |  | 1 |  | 13508.789 |  | 5.979 |  | 0.015 |  |
| BIP |  | 0.038 |  | 1 |  | 0.038 |  | 1.699e -5 |  | 0.997 |  |
| ADHD |  | 6540.835 |  | 1 |  | 6540.835 |  | 2.895 |  | 0.090 |  |
| Residuals |  | 447375.917 |  | 198 |  | 2259.474 |  |  |  |  |  |
|  | | | | | | | | | | | |

| **Basal_Ganglia_ReHo** | | | | | | | | | | | |
| --- | --- | --- | --- | --- | --- | --- | --- | --- | --- | --- | --- |
| **Cases** | | **Sum of Squares** | | **df** | | **Mean Square** | | **F** | | **p** | |
| (Intercept) |  | 1.634e +6 |  | 1 |  | 1.634e +6 |  | 732.650 |  | 1.838e -68 |  |
| SCH |  | 11030.340 |  | 1 |  | 11030.340 |  | 4.946 |  | 0.027 |  |
| BIP |  | 0.059 |  | 1 |  | 0.059 |  | 2.627e -5 |  | 0.996 |  |
| ADHD |  | 2648.296 |  | 1 |  | 2648.296 |  | 1.187 |  | 0.277 |  |
| Residuals |  | 441570.682 |  | 198 |  | 2230.155 |  |  |  |  |  |
|  | | | | | | | | | | | |

| **high_Visual_ReHo** | | | | | | | | | | | |
| --- | --- | --- | --- | --- | --- | --- | --- | --- | --- | --- | --- |
| **Cases** | | **Sum of Squares** | | **df** | | **Mean Square** | | **F** | | **p** | |
| (Intercept) |  | 2.076e +6 |  | 1 |  | 2.076e +6 |  | 1329.878 |  | 8.471e -90 |  |
| SCH |  | 2533.262 |  | 1 |  | 2533.262 |  | 1.623 |  | 0.204 |  |
| BIP |  | 126.890 |  | 1 |  | 126.890 |  | 0.081 |  | 0.776 |  |
| ADHD |  | 1025.198 |  | 1 |  | 1025.198 |  | 0.657 |  | 0.419 |  |
| Residuals |  | 309066.541 |  | 198 |  | 1560.942 |  |  |  |  |  |
|  | | | | | | | | | | | |

| **Language_ReHo** | | | | | | | | | | | |
| --- | --- | --- | --- | --- | --- | --- | --- | --- | --- | --- | --- |
| **Cases** | | **Sum of Squares** | | **df** | | **Mean Square** | | **F** | | **p** | |
| (Intercept) |  | 2.278e +6 |  | 1 |  | 2.278e +6 |  | 1378.423 |  | 3.821e -91 |  |
| SCH |  | 8412.339 |  | 1 |  | 8412.339 |  | 5.090 |  | 0.025 |  |
| BIP |  | 511.390 |  | 1 |  | 511.390 |  | 0.309 |  | 0.579 |  |
| ADHD |  | 5158.229 |  | 1 |  | 5158.229 |  | 3.121 |  | 0.079 |  |
| Residuals |  | 327232.724 |  | 198 |  | 1652.691 |  |  |  |  |  |
|  | | | | | | | | | | | |

| **LECN_ReHo** | | | | | | | | | | | |
| --- | --- | --- | --- | --- | --- | --- | --- | --- | --- | --- | --- |
| **Cases** | | **Sum of Squares** | | **df** | | **Mean Square** | | **F** | | **p** | |
| (Intercept) |  | 3.218e +6 |  | 1 |  | 3.218e +6 |  | 1695.604 |  | 4.955e -99 |  |
| SCH |  | 8137.087 |  | 1 |  | 8137.087 |  | 4.287 |  | 0.040 |  |
| BIP |  | 19.425 |  | 1 |  | 19.425 |  | 0.010 |  | 0.920 |  |
| ADHD |  | 7365.318 |  | 1 |  | 7365.318 |  | 3.881 |  | 0.050 |  |
| Residuals |  | 375786.720 |  | 198 |  | 1897.913 |  |  |  |  |  |
|  | | | | | | | | | | | |

| **post_Salience_ReHo** | | | | | | | | | | | |
| --- | --- | --- | --- | --- | --- | --- | --- | --- | --- | --- | --- |
| **Cases** | | **Sum of Squares** | | **df** | | **Mean Square** | | **F** | | **p** | |
| (Intercept) |  | 3.218e +6 |  | 1 |  | 3.218e +6 |  | 1554.054 |  | 1.090e -95 |  |
| SCH |  | 8128.873 |  | 1 |  | 8128.873 |  | 3.926 |  | 0.049 |  |
| BIP |  | 12.449 |  | 1 |  | 12.449 |  | 0.006 |  | 0.938 |  |
| ADHD |  | 5536.793 |  | 1 |  | 5536.793 |  | 2.674 |  | 0.104 |  |
| Residuals |  | 409993.233 |  | 198 |  | 2070.673 |  |  |  |  |  |
|  | | | | | | | | | | | |

| **Precuneus_ReHo** | | | | | | | | | | | |
| --- | --- | --- | --- | --- | --- | --- | --- | --- | --- | --- | --- |
| **Cases** | | **Sum of Squares** | | **df** | | **Mean Square** | | **F** | | **p** | |
| (Intercept) |  | 2.422e +6 |  | 1 |  | 2.422e +6 |  | 1323.882 |  | 1.251e -89 |  |
| SCH |  | 3593.162 |  | 1 |  | 3593.162 |  | 1.964 |  | 0.163 |  |
| BIP |  | 27.386 |  | 1 |  | 27.386 |  | 0.015 |  | 0.903 |  |
| ADHD |  | 2864.167 |  | 1 |  | 2864.167 |  | 1.565 |  | 0.212 |  |
| Residuals |  | 362266.319 |  | 198 |  | 1829.628 |  |  |  |  |  |
|  | | | | | | | | | | | |

| **prim_Visual_ReHo** | | | | | | | | | | | |
| --- | --- | --- | --- | --- | --- | --- | --- | --- | --- | --- | --- |
| **Cases** | | **Sum of Squares** | | **df** | | **Mean Square** | | **F** | | **p** | |
| (Intercept) |  | 1.479e +6 |  | 1 |  | 1.479e +6 |  | 1111.626 |  | 3.635e -83 |  |
| SCH |  | 1155.965 |  | 1 |  | 1155.965 |  | 0.869 |  | 0.352 |  |
| BIP |  | 8730.251 |  | 1 |  | 8730.251 |  | 6.560 |  | 0.011 |  |
| ADHD |  | 2239.383 |  | 1 |  | 2239.383 |  | 1.683 |  | 0.196 |  |
| Residuals |  | 263516.465 |  | 198 |  | 1330.891 |  |  |  |  |  |
|  | | | | | | | | | | | |

| **RECN_ReHo** | | | | | | | | | | | |
| --- | --- | --- | --- | --- | --- | --- | --- | --- | --- | --- | --- |
| **Cases** | | **Sum of Squares** | | **df** | | **Mean Square** | | **F** | | **p** | |
| (Intercept) |  | 2.941e +6 |  | 1 |  | 2.941e +6 |  | 1350.492 |  | 2.246e -90 |  |
| SCH |  | 5950.320 |  | 1 |  | 5950.320 |  | 2.732 |  | 0.100 |  |
| BIP |  | 160.354 |  | 1 |  | 160.354 |  | 0.074 |  | 0.786 |  |
| ADHD |  | 12704.910 |  | 1 |  | 12704.910 |  | 5.833 |  | 0.017 |  |
| Residuals |  | 431257.649 |  | 198 |  | 2178.069 |  |  |  |  |  |
|  | | | | | | | | | | | |

|  |
| --- |

**Note**: only ROIs with at least 1 significant result were reported
